# A novel hydrogel orthotopic injection model in moderately hypofractionated radiation therapy for prostate cancer: Adaptive degradation and durable imaging

**DOI:** 10.3389/fonc.2022.1077900

**Published:** 2023-01-13

**Authors:** Hao Yu, Cheng Wang, Lingyun Wu, Ziyang Zhou, Yiqi Wang, Wenxiang Li, Huili Yuan, Zeyi Lu, Danfang Yan, Si Chen, Xu Wang, Senxiang Yan

**Affiliations:** ^1^ Department of Radiation Oncology, The First Affiliated Hospital, School of Medicine, Zhejiang University, Hangzhou, Zhejiang, China; ^2^ College of Materials Science & Engineering, Zhejiang University of Technology, Hangzhou, Zhejiang, China; ^3^ Department of Urology, Sir Run Run Shaw Hospital, School of Medicine, Zhejiang University, Hangzhou, Zhejiang, China

**Keywords:** prostate cancer, hydrogel spacer, adaptive degradation, durable imaging, moderately hypofractionated radiotherapy (MHRT)

## Abstract

**Purpose:**

Moderately hypofractionated radiotherapy (MHRT) holds an important position in prostate cancer management. Existing hydrogel spacers can protect the rectum from radiation damage, but need improvement. We explored the application of a novel hydrogel in MHRT with adaptive degradation and durable imaging functions.

**Methods and materials:**

The hydrogels were irradiated with 6MV x-ray to detect the radio-resistance property. Male SD rats (n=45) underwent hydrogel injection between the prostate and rectum. CT was used for investigating the novel spacer’s degradation and imaging functions over three months. The hydrogel’s radiation-attenuation properties and biocompatibility were further assessed.

**Results:**

Hydrogel weight and volume remained stable for six weeks post-injection. After MHRT ended, the hydrogel showed accelerated degradation characteristics and remained in the body for at most three months. CT values of hydrogels exceeded 300 Hounsfield units (HU) throughout treatment, significantly higher than in surrounding normal tissues. A significant dose drop behind the hydrogel was observed post-implantation. Biocompatibility tests of hydrogel found it safe enough for living organisms.

**Conclusions:**

The novel hydrogel application was fully adaptable to prostate cancer MHRT modalities, largely stable during treatment, rapidly degraded after radiotherapy ended, and consistently maintained superior imaging performance and biocompatibility. This novel spacer will be an effective tool in the era of hypofractionated radiotherapy.

## 1 Introduction

Prostate cancer is the second most common cancer in men, accounting for 7% of newly-diagnosed cancers worldwide. Nearly 1.3 million new cases were diagnosed and more than 350,000 prostate cancer-related deaths occurred globally in 2018, making it one of the recognized leading causes of cancer-related death in men (1). Radiotherapy (RT) holds an extremely high position in the management of prostate cancer and is curative in 60% of men with localized prostate cancer (2). For localized prostate cancer, the efficacy of RT is similar to that of surgery, but RT is less invasive (3). However, anatomical proximity to the prostate makes the anterior rectal wall susceptible to radiation damage (4). The increased volume of rectal irradiation leads to a higher risk of developing early and late gastrointestinal complications. Conventionally-fractionated RT and hypofractionated RT are commonly used modalities in the clinic. Moderately hypofractionated RT (MHRT), 2.1–3.5 Gy per fraction, lasting 10 minutes per day, five days per week, for about 4–6 weeks (5). Compared with conventional modalities, MHRT increases the fractionated dose to tumor tissue and shortens the overall treatment time, improving patient comfort and convenience as well as the cost-effectiveness of treatment. Multiple clinical studies have clarified the non-inferiority of MHRT for prostate cancer relative to conventionally fractionated RT (6–9). However, a large, randomized phase-3 trial showed significantly higher levels of acute bowel symptoms with MHRT in patient-reported outcomes (10). Thus no matter which RT modality is used, damage to the rectum is difficult to avoid. The spacer technique is promising because it implants a biomaterial into the underlying space between the prostate and rectum, diverting the anterior rectal wall away from the high-dose zone and reducing rectal damage (11).

**Figure 1:**
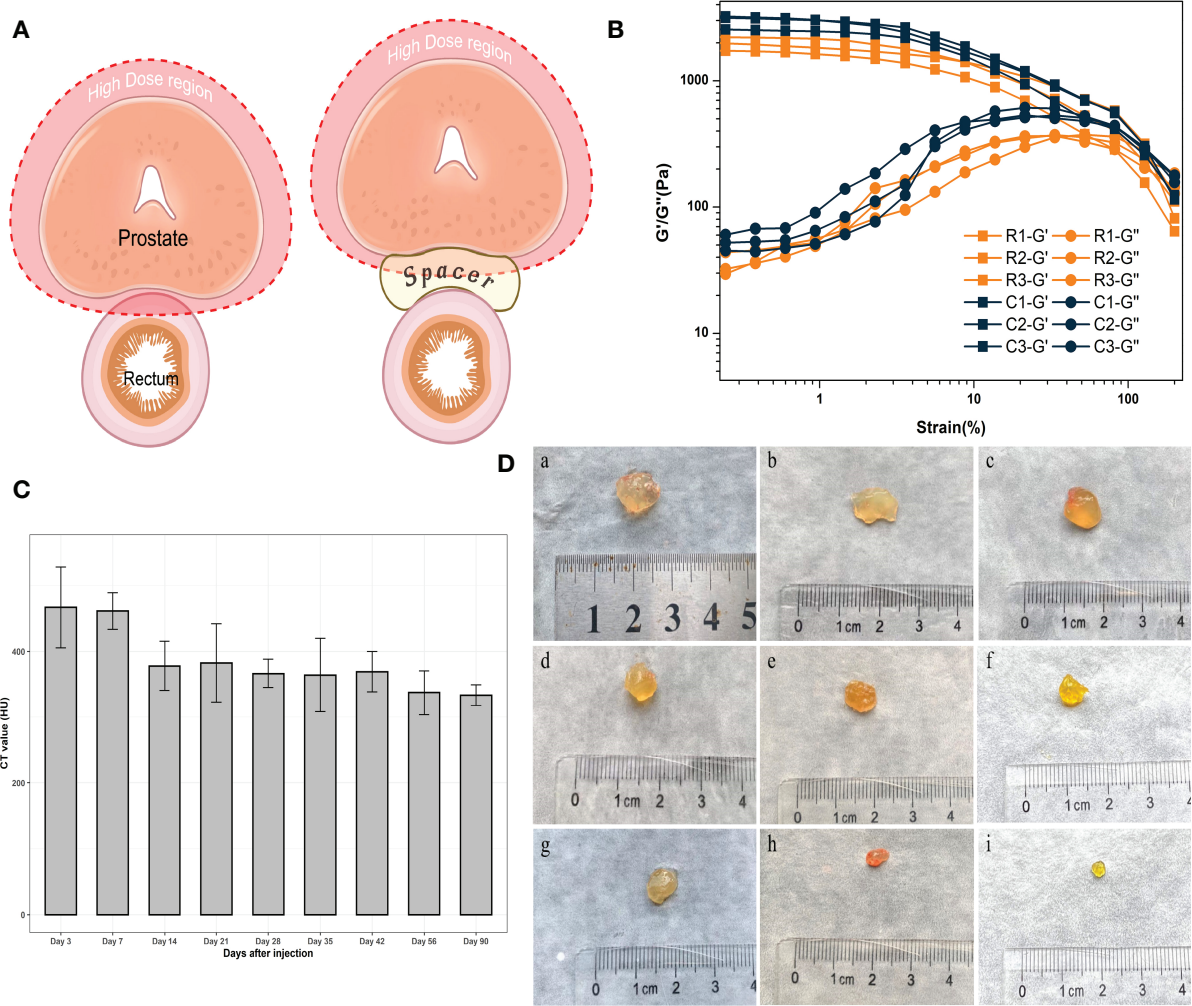
**(A)** The application of the spacer in radiotherapy for prostate cancer. (Created with BioRender.com.) **(B)** Rheology study of hydrogels exposed in radiation. **(C)** CT values of hydrogels. **(D)** Residual hydrogels obtained from rats; “a” to “i” represent 3, 7, 14, 21, 28, 35, 42, 56, 90 days after injection, respectively.

Various spacer materials have been evaluated in recent years, such as hydrogels (4, 12), collagen (13), and inflatable balloons (14) (**Figure 1A**). However, the inflated balloon is not degradable *in vivo* and needs to be surgically removed (14); collagen is not sufficiently supportive and degrades too rapidly (13). A prospective, multicenter, randomized controlled trial demonstrated a significant reduction in mean rectal volume receiving at least 70 Gy (V70) (3.3% vs. 12.4%, P <.0001) and late (3-15 months after radiation) rectal toxicity (2.0% vs. 7.0%, P = .04) in the perirectal hydrogel spacer group compared with the control group (4). This result makes injectable hydrogels the most attractive materials to protect the rectum in prostate cancer RT based on their high shape stability and biological affinity. However, some existing hydrogels still have some disadvantages. For example, polyethylene glycol (PEG) hydrogels begin to degrade 3 months after implantation, and complete dissolution requires half a year (15, 16). A longer retention of the implant in the body may increase potential rare side effects, such as pulmonary embolism, rectal ulcers, and fistula formation (17). Therefore, the novel hydrogel spacer was designed to be suitable for MHRT modalities with short treatment cycles to enhance biological compatibility. Meanwhile, implants can undergo degradation as soon as possible after the end of RT to increase patients’ comfort.

A higher gradient of dose distribution requires precise determination of the target volume location, or the target may be “precisely” missed (18). The intra- and inter-fraction variation of gross tumor volume (GTV) position in RT may lead to treatment-related risks. Moreover, hydrogels cannot be distinguished using computed tomography (CT) as they have similar density to soft tissues, which increases the uncertainty of the treatment. However, because of the drug burst effect, most current methods of adding contrast agents directly to hydrogels cannot maintain imaging function for minimum four-weeks time requirement for MHRT of prostate cancer (19). Although a few hydrogels with sustainable imaging functions can retain imaging function throughout the treatment, some hydrogels have initial CT values of only about 140 HU, and the imaging ability of hydrogels decreases obviously over time (19). Their imaging performance is worse at the first cone beam CT (CBCT) verification than at the first injection.

In our previous research, we developed a new, injectable hydrogel with characteristics of rapid gelation, adequate mechanical properties, and controlled degradation (20). This study mimics the natural environment between the prostate and rectum, demonstrating the volume stability and durable imaging function of novel hydrogel spacers during prostate cancer MHRT. Hydrogels possess properties of accelerated degradation after the end of MHRT, which not only fulfills the needs for treatment, but also minimizes the potential risks. Meanwhile, CT values remained above 300 HU throughout the treatment, meaning the hydrogel can be sufficiently visualized in CT scans without requiring MRI validation. Finally, we tested the biocompatibility of the hydrogel and found it sufficiently safe for living organisms. This work provides evidence of rectal protection under MHRT for prostate cancer and sheds new light on organ protection in the era of hypofractionated RT in the future.

## 2 Materials and methods

### 2.1 Radiation-tolerance assay

The hydrogels were irradiated with 6 MV x-ray (3 Gy per fraction). The doses of the experimental groups were 0 Gy, 18 Gy in 6 fractions, 36 Gy in 12 fractions, 54 Gy in 18 fractions, 72 Gy in 24 fractions, and 81 Gy in 27 fractions, respectively. Set up corresponding control groups for each radiation experimental group, where the control group received no radiation and the remaining conditions were the same as the radiation group. An Anton-paar MCR 302 Rheometer was used to measure the morphology behavior of hydrogels, characterize the gel strength, and assess whether the hydrogel structure was damaged.

### 2.2 Preparation of experimental animals

We used 55 male Sprague Dawley (SD) rats (10 weeks old, 350–400 g each); these were randomly divided into 11 groups: nine experimental groups, and one group each for the negative and positive controls. Rats were kept in sterile conditions and provided with autoclaved food and sterile water. All the experimental procedures were approved by the Lab of Animal Experimental Ethical Inspection of the First Affiliated Hospital, Zhejiang University School of Medicine (Reference Number: 2022-1509). The experiment was also carried out according to the scheme approved by the Zhejiang University of Technology.

### 2.3 Hydrogel implantation surgery

The animals all underwent open surgery after anesthesia. Rats were fixed on the operating table after anesthesia and hair was removed from their abdomens. After disinfection, the skin and tissues were incised in the middle of the lower abdomen, and the bladder, prostate, and rectum were identified after entering the abdominal cavity. The syringe needle was carefully inserted between the prostate and rectum for hydrogel injection. The designed volume of the hydrogel is 0.25–0.30 ml per rat. The negative control group was injected with sterile saline following laparotomy. The experimental procedures were all performed under sterile conditions. The skin was sutured postoperatively with sterilized sutures. The animals were housed in an environment of 24 ± 2°C, with 12 hour alternating light and dark periods and free access to water and food.

### 2.4 CT scan and specimen retrieval

Members of the experimental group were randomly selected for CT scan (SOMATOM Definition AS Open, Siemens, Hamburg, Germany) and dissection at 3, 7, 14, 21, 28, 35, 42, 56, and 90 days after surgery. *In vivo* variation of volume and CT intensity was evaluated by medical imaging software (MIM, Cleveland, USA). The rats were sacrificed after CT scanning. The hydrogels were removed intact and weighed. The organs, blood, and semen were obtained for analysis.

### 2.5 *In vivo* radiation planning

The hydrogels and surrounding normal tissues were contoured using the Eclipse treatment planning system (Varian Medical Systems, Inc., California, USA) to obtain the CT volumes. Target volumes were contoured consistently to avoid bias. At the same time, the parameters involved in the plan formulation were kept consistent. Used single field irradiation (4 MeV electron beam, SSD: 98cm, radiation field size: 6*6cm, dose: 300cGy/fraction) to verify the performance of radiation attenuation after hydrogel implantation. Considering the short distance from the subcutaneous area of rats to the prostate, compared with the depth of maximum dose of 6MV photon beam, 4-6MeV electron beam is more suitable for simulating the clinical dose attenuation.

### 2.6 Cytotoxicity assay

Mouse fibroblast cells (L929) were obtained from Procell (Wuhan, China) and were cultured in Special Culture Medium (Procell, Wuhan, China), supplemented with Minimum Essential Medium (MEM), 10% fetal bovine serum (FBS), and 1% Penicillin/Streptomycin solution. Cultured cells were maintained at 37°C in an atmosphere containing 95% air and 5% carbon dioxide. The cytotoxicity of the hydrogels was investigated using the MTT assay. L929 cells were first seeded in a 96-well plate at a density of 1*104/well; after incubation in a Special Culture Medium for 24 h the medium was discarded, and different concentrations of hydrogel extracts were added to each well in the experimental group. Hydrogel extracts were obtained by soaking the hydrogel in Special Culture Medium and standing for 24 h in a 37°C incubator. Special Culture Medium was used as a negative control, while phenol solution with a concentration of 0.64% was added to the positive control group. The incubation was continued for 4 hours and then replaced by MTT solution with a concentration of 1 mg/ml. After continuing the incubation for 2 hours, the MTT solution was aspirated, and isopropanol was added. All of the above solutions were filtered to remove bacteria. Absorbance was recorded at 570 nm using a microplate reader (MD SpectraMax i3x, USA), and relative cell survival was calculated with the reported protocol.

### 2.7 Genotoxicity

The bone marrow micronucleus assay, which can determine whether a substance damages the chromosomes or the mitotic apparatus of red blood cells, has been shown to be an effective method to detect the genotoxicity of certain materials. We randomly divided 25 SPF-grade Kunming mice (6–8 weeks old, 18–20 g each) into five groups—three experimental groups, one negative control group, and one positive control group—of five mice, each. The hydrogel extracts were obtained by Soxhlet extraction. The three experimental groups were injected intraperitoneally with 20 ml/kg of hydrogel extract at concentrations of 100%, 80%, and 50%, respectively. The negative control group was injected with an equal volume of saline. The positive control group was injected with cyclophosphamide at a dose of 60 mg/kg. The injection was repeated once after an interval of 24 h. Mice were sacrificed 6 h after the last injection, and their femurs were removed. The bone marrow cavity of the femurs was washed with 1 ml of sterile fetal bovine serum. Bone marrow cells were collected by washing the femoral bone marrow cavity with 1 ml of sterile fetal bovine serum. These sera were smeared, and at least three smears were prepared for each mouse. After air drying for 24 h, methanol was used for dehydration fixation, followed by Giemsa staining. An Olympus CX31 Microscope (Olympus Co., Tokyo, Japan) was used to examine micronuclei in polychromatic erythrocytes (PCEs). We counted the number of PCEs and normochromatic erythrocytes (NCEs) per 1000 RBCs and calculated the PCE/NCE ratio.

### 2.8 Reproductive toxicity

#### 2.8.1 Testosterone analysis

Rats in the experimental and negative control groups were treated as above. The five rats in the positive control group received an intraperitoneal injection of cyclophosphamide at a dose of 100 mg/kg. Animal blood was collected during dissection, and samples were centrifuged for 10 minutes at 4000 × g to collect the serum. Serum testosterone levels were detected using the Rat Testosterone ELISA Kit (Fankewei, Shanghai, China).

#### 2.8.2 Sperm motility and quantity

Rats were sacrificed at corresponding time points. The left epididymis was weighed and placed into a clean, flat dish. Then, 3 ml of 1640 culture solution, preheated to 37°C, were added to the culture dish, and the epididymis was completely shredded. The culture dish was put into a 37°C shaker for 5 minutes to achieve sperm suspension. The sperm suspension was mixed with normal saline in a 1:50 ratio, then 10 µl of the diluted sperm suspension was taken and added to the hemocytometer. After resting for 5 minutes, the hemocytometer was placed under a microscope (Nikon ECLIPSE Ti-S, Japan) to count the number of non-linearly motile spermatozoa (including dead spermatozoa, swinging rotary motile spermatozoa). Then, the hemocytometer was put into the 120°C dry oven for 5 minutes, and the sperm were killed and counted to find the total number of sperm per gram in the epididymis. Sperm motility = (total sperm count − non-linearly motile sperm count)/total sperm count * 100%.

#### 2.8.3 Sperm-malformation analysis

The right epididymis was put into 37°C pre-warmed normal saline, sheared, and filtered in four layers. The filtrate was collected for smear, and 500 sperm per animal were observed by microscope after methanol fixation for sperm-malformation analysis.

#### 2.8.4 Reproductive organ weight and organ coefficient

The bilateral testes and epididymides of each rat were weighed separately. The weight coefficient of the testis = bilateral testicular weight/body weight. The weight coefficient of the epididymis = bilateral epididymis weight/body weight.

### 2.9 Blood indexes analysis

Animal blood was collected during dissection. Samples were centrifuged for 10 minutes at 4000 × g to collect the serum. Red blood cells (RBC), hemoglobin (HGB), white blood cells (WBC), and platelets (PLT) were detected using a whole-blood analyzer. The alanine aminotransferase (ALT), aspartate aminotransferase (AST), creatinine (Cr), and the electrolytes, such as sodium (Na) and potassium (K) were tested using an automated chemistry analyzer (FUJI DRICHEM 4000ie, Fujifilm Corporation, Tokyo, Japan).

### 2.10 Histopathological analysis

The main organs of rats, including: heart, lung, liver, kidney, spleen, testis, epididymis, and the hydrogel-adjacent rectal, prostate, and bladder tissues were collected and evaluated by histopathological tests. Tissues were embedded in paraffin, sectioned at 5 mm thickness, and stained with hematoxylin-eosin (H & E). An experienced pathologist observed and confirmed the randomly numbered sections for any damage.

## 3 Results

### 3.1 Radiation tolerance assay

Hydrogels were allocated to hypofractionated RT of 81 Gy in 27 fractions over 6 weeks. The total experimental dose greatly exceeded the clinically-practical application of hypofractionated RT. The strength of the hydrogel in the experimental group was not significantly decreased compared to the control group, which confirmed that the hydrogel network was not damaged after RT (**Figure 1B**). G’ is the energy storage modulus, which represents the elasticity of the hydrogel; G’’ is the energy loss modulus, which represents the viscosity of the hydrogel. The intersection of these two is a measure of whether the hydrogel network is completely disrupted. There was no significant decrease in the mechanical strength (elasticity) of the gel in the radiotherapy group compared with the control group, and the point of disruption of the hydrogel was similar in both groups. Therefore, it was confirmed that the hydrogels could tolerate radiotherapy irradiation. This positive result was a prerequisite to ensuring that the hydrogel continued to support the spacing during treatment.

### 3.2 *In vivo* long-lasting imaging function and degradation

The purpose of this study was to develop a hydrogel with long-lasting imaging function and degradation conforming to the MHRT timeline of prostate cancer. CT scanning of the rats at each time point revealed that the hydrogel was clearly intensified on the images, sharply demarcated from the surrounding normal tissue, and still had high imaging intensity 90 days post-injection. We used CT values to describe the imaging properties of the hydrogels (**Figure 1C**). After injection, the CT values were all above 300 HU, and the radiopacity was significantly higher than that of the surrounding normal tissues (approximately 30–75 HU for prostate, seminal vesicles, and rectum).

**Figure 2:**
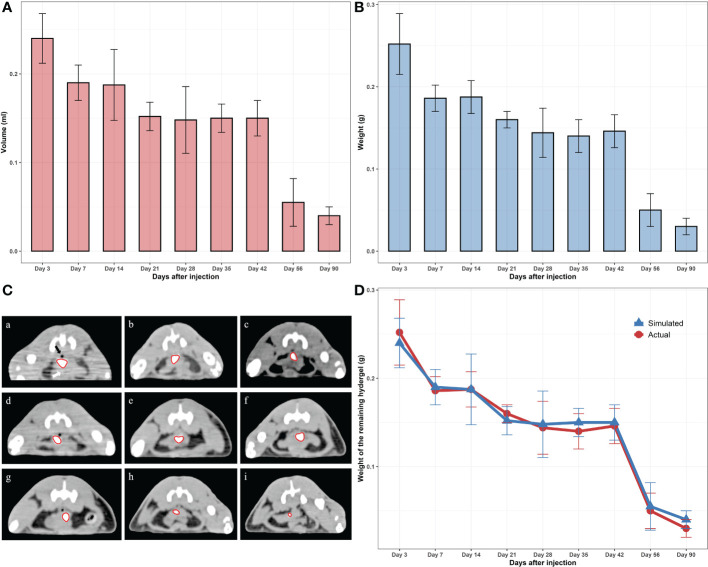
**(A)** Virtual (contoured) volumes of the residual hydrogels. **(B)** Weights of the actual residual hydrogels. **(C)** CT photos of the remaining hydrogels (scale bar = 3 cm); “a” to “i” represent 3, 7, 14, 21, 28, 35, 42, 56, 90 days after injection, respectively. **(D)** Comparison of the real and virtual weights of the hydrogels.

MIM software (MIM Software Inc., Cleveland, USA) was used to evaluate the changes of hydrogels. CT scanning was performed at each time point within 90 days after injection to draw the contour of the hydrogel area and calculate the hydrogel volume with a three-dimensional configuration simulation (**Figure 2C**). After CT scanning, the rats were sacrificed, and the hydrogel was taken out as a single piece (**Figure 1D**). Because the density of the hydrogel was similar to that of water (the density of the hydrogel was approximately equal to 1g/cm^3^), contouring the hydrogel on CT and calculating the volume yielded an approximate simulated weight that could be contrasted with the actual weight of the hydrogel after it was removed from the body (**Figures 2A, B**). As expected, we found an apparent similarity between the actual and the simulated weights within three months (**Figure 2D**), indicating that the residual volume and the weight of hydrogels *in vivo* can be accurately estimated by noninvasive detection means such as CT scans in clinical work. Meanwhile, hydrogel degradation in the space between the prostate and rectum occurs at a specific pace. The degradation rate was faster within 7 days after injection, probably because there was a slight loss of the freshly injected hydrogel in the interstitial space. Between 7 and 42 days after injection, the degradation rate approached a plateau during which both the volume and weight of the hydrogels remained stable. Once 42 days had passed, the hydrogel again entered a rapid degradation period, and only a small part remained at 90 days. This illustrated that the novel hydrogel was well suited for MHRT of prostate cancer to keep the position of the rectum and prostate relatively consistent during the treatment, making the irradiated area more stable and significantly reducing the treatment-associated risk.

### 3.3 Radiation dose fall-off ability

**Figure 3:**
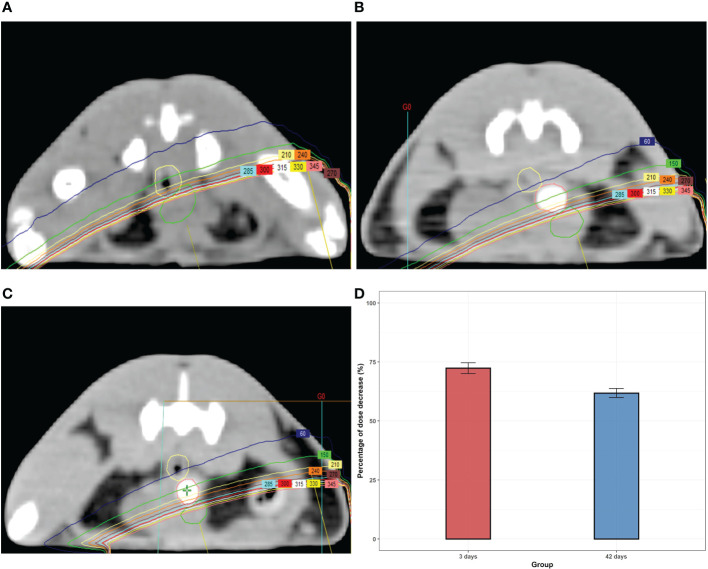
The dose distributions from the axial CT section of rats with or without **(A)** hydrogel at day 3 **(B)** and day 42 **(C)**. **(D)** The percentage of the dose-drop at day 3 and day 42.

Whether hydrogels can realistically reduce the radiation dose of the anterior rectal wall needs further exploration. We used the Varian Eclipse Treatment Planning System (Varian Medical Systems, Palo Alto, CA) to deliver a single-fraction RT plan to the target location. The dose distributions from axial CT sections are present in **Figure 3**. An obvious dose drop behind the hydrogel was observed after implantation. The average percentage of dose reduction at the anterior rectal wall was around 70% compared with the non-implant group both on days 3 and 42 after injection.

### 3.4 Cytotoxicity and genotoxicity

MTT assay is widely used to measure cell viability and drug cytotoxicity. In the cytotoxicity test, we set up five experimental groups at different concentrations, the highest concentration was 100%, after which each group was diluted 0.75–fold; the final concentrations of the experimental groups were 100%, 75%, 56%, 42%, 32%, respectively. Negative and positive control groups were set up simultaneously. They were not considered toxic to L929 cells in all experimental groups, even at 100% concentrations. Our results showed that the novel hydrogels did not exhibit cytotoxic potential (**Figure S1**).

The bone marrow micronucleus test is a standard method to detect chromosomal damage and chemical toxicants that interfere with cell mitosis. Micronuclei are generally believed to result from exposure of cells to chromosomal breakage agents or spindle poisons. The presence of micronuclei is expressed as ‰ MNPCE. The effects of the hydrogel leaching solutions on bone marrow micronuclei in mice are summarized in **Table 1**. The results showed that the positive control significantly improved ‰ MNPCE compared to the negative control (P < 0.01), whereas the differences between each experimental group and the negative control group were not significant (P > 0.05). This assay can also reflect the cytotoxicity of tested materials through the PCE/NCE ratio. If the normal proliferation of myeloid cells is affected by toxic chemicals, the PCE/NCE ratio will decrease. The PCE/NCE ratio was not significantly reduced in the experimental group compared to the negative control group (P > 0.05). The novel hydrogel showed no genotoxicity in experimental animals in line with the ‰ MNPCE value and the PCE/NCE ratio.

**Table 1:** Genotoxicity of the novel hydrogel.

Group	‰MNPCE	Ratio PCE/NCE
100%	1.20	0.98 ± 0.06
80%	1.40	0.97 ± 0.06
50%	1.40	1.01 ± 0.06
Negative control	1.00	1.01 ± 0.03
Positive control	20.40***	0.96 ± 0.04

*** P<0.005.

### 3.5 Reproductive toxicity

**Figure 4:**
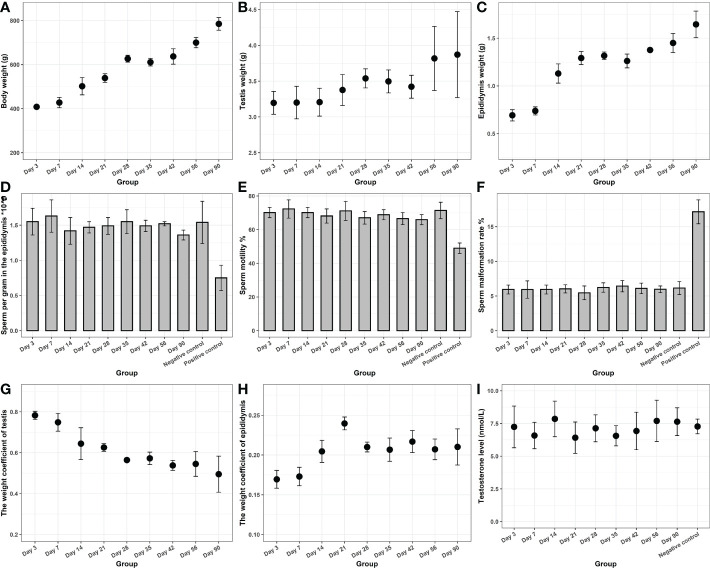
Reproductive toxicity of the novel hydrogel. The body weight **(A)**, epididymis **(B)**, and testis weight **(C)** of rats. **(D)** The epididymal sperm count. **(E)** The sperm motility. **(F)** The sperm malformation rate. **(G)** The weight coefficient of the testis. **(H)** The weight coefficient of the epididymis. **(I)** Testosterone levels.

The results of the reproductive toxicity experiments are presented in **Figure 4**. The body weight, epididymis, and testis weight of rats increased gradually over the study period. The weight coefficient of the epididymis increased and then leveled off, whereas that of the testis gradually decreased. A possible reason for this is that with the increase in feeding days, the rate of body weight gain was significantly higher than that of the testis. Testosterone levels in each experimental group were not significantly different from those of the negative control group (P > 0.05). The epididymal sperm count, sperm motility, as well as sperm malformation rate in each experimental group were significantly higher than those in the cyclophosphamide group (P < 0.05), but were not different from those in the control group (P > 0.05). All of the results confirmed that the novel hydrogels had no potential reproductive toxicity.

### 3.6 Hematological and histological evaluation

**Figure 5:**
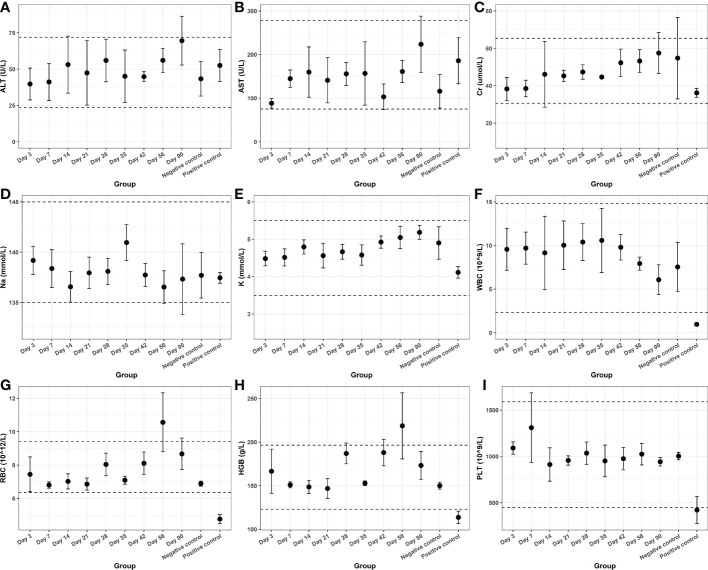
The hematological indexes of **(A)** ALT, **(B)** AST, **(C)** Cr, **(D)** Na, **(E)** K, **(F)** WBC, **(G)** RBC, **(H)** HGB and **(I)** PLT from blood samples.

**Figure 6:**
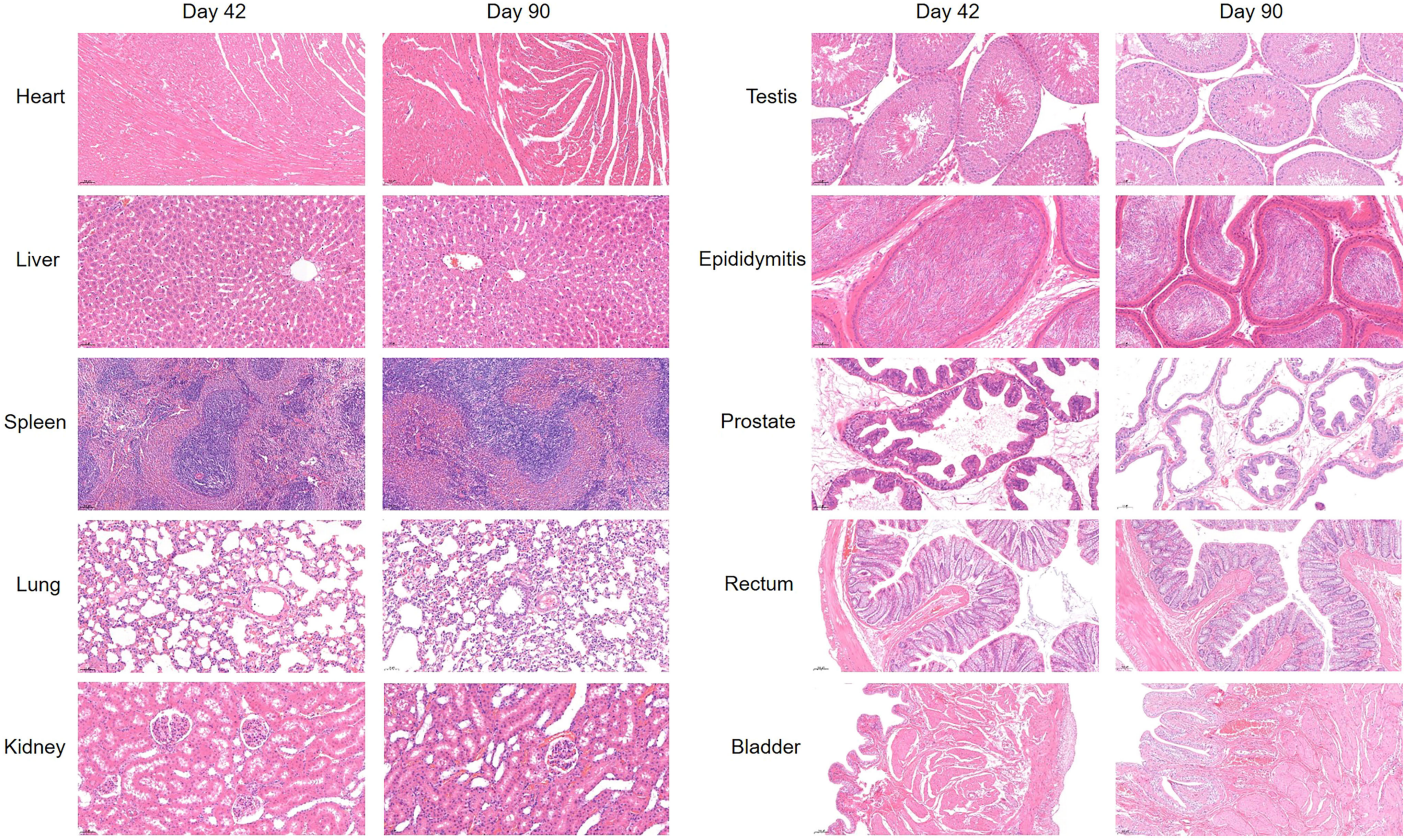
H & E-stained images of the heart, lung, liver, kidney, spleen, testis, epididymis, and hydrogel-adjacent rectal, prostate, and bladder tissues at day 42 and day 90.

Finally, we tested the blood and organ tissues of each group of experimental rats. Serum detection indicators include ALT, AST, Cr, Na, and K. Whole-blood test indicators included WBC, RBC, HGB, and PLT. The results showed no additional side effects (**Figure 5**). Meanwhile, the heart, lung, liver, kidney, spleen, testis, epididymis, and hydrogel-adjacent rectal, prostate, and bladder tissues of each rat were examined histopathologically by hematoxylin-eosin staining, and the results are shown in **Figure 6**. No noticeable morphological changes were observed.

## 4 Discussion

In this study, we demonstrated the volumetric stability and durable imaging function of novel hydrogel spacers during prostate cancer MHRT, which greatly reduced the dose received by the rectum and improved the precision of target-volume location. The hydrogel was tested for both radioresistant and radio-attenuating properties. It can maintain stable rheology properties under irradiation at far higher doses than are used in actual clinical radiation, and also keep a higher radiopacity at the end of hypofractionated treatment. In addition, CT values remained above 300 HU throughout the treatment, which means the hydrogel can be sufficiently visualized in CT scans without requiring MRI validation. Finally, we tested the biocompatibility of the hydrogel and found it safe enough for living organisms.

Cell death following ionizing radiation is associated with a linear quadratic model that describes the relationship between cell survival, total dose, and fraction. Use the α/β Ratios to characterize the tissue response to the dose fractionation. It has been reported that the α/β Ratio of most tumors is approximately 10 Gy, whereas that of prostate cancer is 0.9–2.2 Gy (21). Tissues with lower α/β Ratios demonstrate greater sensitivity to hypofractionated RT. MHRT is widely considered a viable alternative to conventional RT in patients with localized prostate cancer. The accumulated evidence suggests that the use of MHRT can be recommended regardless of cancer risk group (22). Several studies have demonstrated no difference in toxic effects between the hypofractionated and conventional groups (6, 23, 24). However, a recent meta-analysis suggested that risk of grade 2, or worse, acute gastrointestinal (GI) adverse events in the MHRT group was increased by 9.8% (95% CI [4.8–14.7%]) (25). HYPRO was a randomized, phase 3 trial consisting of 820 patients (410 in both groups) receiving 78 Gy in 39 fractions or 64.6 Gy in 19 fractions. After 5 years of follow-up, studies failed to demonstrate non-inferiority for cumulative late GI toxicity of hypofractionated RT, and the hazard ratio (HR) for cumulative grade≥2 late GI toxicity was estimated to be 1.19 (90% CI [0.93–1.52]) (8). Taken together, we can reasonably assume that hydrogels which significantly reduce rectal toxicity in conventional RT may be more practical in MHRT.

Existing hydrogel products for prostate cancer radioprotection stay in the body for half a year, exceeding the duration of the RT course, which may increase the incidence of certain uncommon complications, such as pulmonary embolism, rectal ulcers, colostomy, anaphylactic events or rectal-wall injection (26). The novel hydrogel remained stable in weight and volume for one-and-a-half months after injection. It maximized the stabilization of the relative positions of the prostate and rectum during RT, enhancing the precision of the radiation target volume. Then, the novel hydrogel degraded rapidly after the end of RT, decreasing the retention time of the implant *in vivo*, reducing potential side effects, and increasing patient comfort. However, this also carries the risk that 6 weeks is perhaps too short, allowing very little leeway for treatment delays or other unanticipated complications. This may require further improvements to the new hydrogel to reduce this risk. Meanwhile, because the injected hydrogel was large in volume and pushed the rectum entirely away from the prostate, its ability to attenuate radiation was strong, reaching about 70% in the simulated state. In human experiments, the volume of PEG hydrogel injected between Denonvilliers’ fascia and the anterior wall of the rectum is typically ten milliliters. Its diameter is much smaller than that of the rectum and prostate, and it can reduce the rectal dose by 25% (17). The actual radiation dose fall-off ability of this novel hydrogel needs to be confirmed by clinical studies.

In clinical work, repeated CBCT was performed to ensure target volume positions. Some of the currently commercially available hydrogel products have low contrast in CT scans compared to soft tissues and may not be accurately measured by clinicians. This hydrogel may require MRI for further adequate visualization (27). Therefore, MRI scans are often helpful in delineating the spacer in addition to the CT scan. However, if the radiation oncology center cannot efficiently and reliably perform an MRI scan, or if the patient has an absolute contraindication to an MRI, such hydrogels will be limited. In addition, the inclusion of an MRI as part of a treatment plan increases costs and inconvenience. A few imageable hydrogels also have problems, such as too short an imaging duration (27), insufficient imaging intensity, etc. Improved contrast with soft tissue by covalently bonding iodine rather than physical mixing on the hydrogel allows clinicians to adequately visualize spacers in CT scans, obviating the need for MRI to aid in target volume delineation. Another advantage of the novel material is its visualization without attenuation throughout the treatment period. Further biocompatibility tests demonstrated the biosafety of the novel hydrogel. In combination with the above results, applying the novel hydrogel spacer in patients undergoing prostate cancer RT is promising.

## 5 Conclusion

We showed that the novel hydrogel application was fully adaptable to prostate cancer MHRT modalities, largely remained stable during treatment, and rapidly degraded after the end of RT; and that it consistently maintained superior imaging performance and biocompatibility. This novel spacer appears to be an effective tool in the era of hypofractionated RT.

## Data availability statement

The original contributions presented in the study are included in the article/**Supplementary Material**. Further inquiries can be directed to the corresponding authors.

## Ethics statement

The animal study was reviewed and approved by the Lab of Animal Experimental Ethical Inspection of the First Affiliated Hospital, Zhejiang University School of Medicine (Reference Number: 2022-1509). The experiment was also carried out according to the scheme approved by the Zhejiang University of Technology.

## Author contributions

Study conception or design: HY, CW, LW, SY, SC, and XW. Project administration: SY, SC, and XW. Acquisition, analysis, or interpretation of the data: HY, CW, ZZ, YW, WL, and HLY. Visualization: HY, CW, and ZL; Writing and revision of the manuscript: HY, CW, and DY. All authors contributed to the article and approved the submitted version.

## Supplementary material

Supplementary Figure 1 Cytotoxicity of the novel hydrogel.
