# Supplementary figures and images for: A novel hydrogel orthotopic injection model in moderately hypofractionated radiation therapy for prostate cancer: Adaptive degradation and durable imaging
Source: Front Oncol. 2023 Jan 13;12:1077900. doi: 10.3389/fonc.2022.1077900 (PMC9880553; doi:10.3389/fonc.2022.1077900)

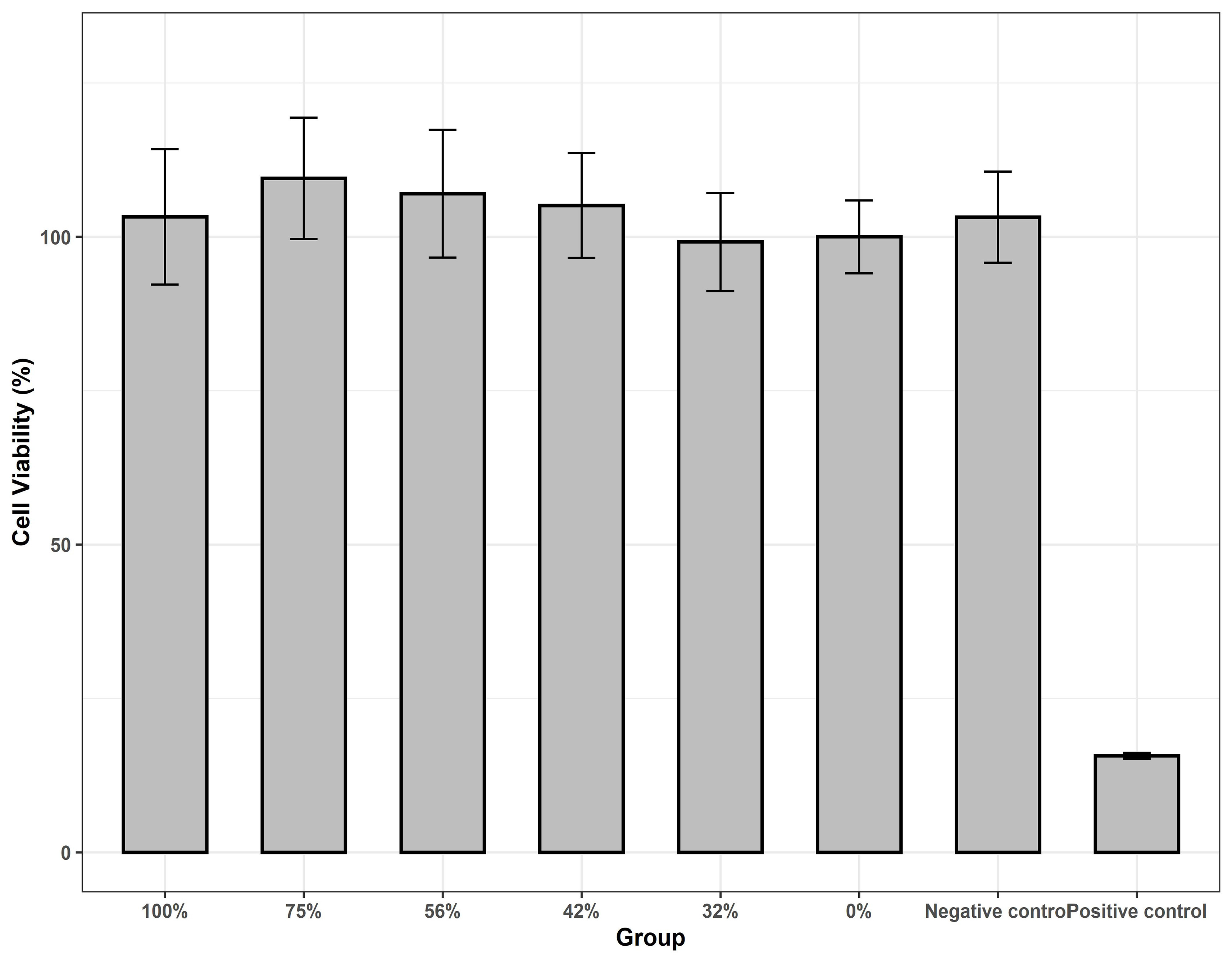

Supplement: Supplementary Figure 1 — Cytotoxicity of the novel hydrogel. [file Image_1.jpeg]
